# Trace Element Intake from Dairy-Free Infant Porridges and Its Nutritional and Safety Implications for Infants Aged Six Months and Older

**DOI:** 10.3390/nu18020333

**Published:** 2026-01-20

**Authors:** Zuzanna Chronchol, Agata Witczak, Kamila Pokorska-Niewiada

**Affiliations:** Department of Toxicology, Dairy Technology and Food Storage, West Pomeranian University of Technology in Szczecin, 71-454 Szczecin, Poland; cz49796@zut.edu.pl (Z.C.); awitczak@zut.edu.pl (A.W.)

**Keywords:** baby food, infant porridges, trace element, harmful elements, health risk, essential elements, health benefits

## Abstract

**Background/Objectives**: Following the cessation of breastfeeding, cereal-based complementary foods are commonly introduced into the diet of infants. Among these products, dairy-free infant porridges constitute an important component of early complementary feeding. This study aimed to evaluate dietary exposure to selected essential (Zn, Fe, Mn, and Cu) and potentially toxic (Pb, Cd, and Hg) trace elements resulting from the consumption of dairy-free infant porridges by children aged six months and older. Products with different cereal compositions available on the Polish market were analyzed. **Methods**: Trace element concentrations were determined after microwave-assisted digestion using inductively coupled plasma atomic emission spectrometry (ICP-AES) and atomic absorption spectrometry for mercury (Hg-AAS). **Results**: A single recommended serving of dairy-free infant porridge contributed to the intake of essential trace elements, providing approximately 50% of the RDA for copper, 21% for zinc, 15% of the AI for manganese, and 5.7% of the RDA for iron. The concentrations of potentially harmful elements were low (Pb: 0.002–0.004 mg/kg; Cd: <0.001–0.003 mg/kg; Hg: <0.001–0.001 mg/kg). The estimated daily intake of these elements did not exceed 0.01 µg/kg body weight per day. **Conclusions**: Dairy-free infant porridges may contribute to the intake of essential trace elements in infants, while exposure to lead, cadmium, and mercury appears to remain low when products are consumed according to recommended serving sizes.

## 1. Introduction

The first 1000 days of a child’s life, from conception to their second birthday, are a period of intense development and growth. This is an extremely important time for the future health of every human being [1,2,3]. A child’s development is closely linked to proper nutrition. It is recognized that at six months of age, feeding a child only breast milk may not meet their energy and nutrient requirements. The European Society for Pediatric Gastroenterology Hepatology and Nutrition (ESPGHAN) recommends that the gradual introduction of liquids and solid foods other than breast milk or infant formula into an infant’s diet should begin between 17 and 26 weeks of age [4]. At this time, it is important to introduce complementary foods to provide a varied, nutrient-rich diet adapted to the stage of development of the infant. Examples of such foods include infant porridges. Infant porridges are defined as processed cereal products that play a key role in expanding infants’ diets and gradually preparing their bodies to consume foods of varying consistencies. Due to their composition, they are divided into two groups: processed cereal products with added high-protein ingredients (prepared by adding water or other non-protein liquids) and products without added high-protein ingredients (prepared by adding milk or other liquids) [5,6]. These porridges differ in the percentage composition of basic nutrients such as protein, fat, and carbohydrates [6,7]. Porridge not only provides essential nutrients, but also promotes the positive development of the gut microbiome [8,9]. Grains such as millet and buckwheat are naturally gluten-free and are therefore often recommended for children with food intolerances such as celiac disease. In addition, these grains may also be beneficial in preventing childhood obesity, as introducing fiber-rich foods can help regulate appetite and promote healthy eating habits from an early age [10]. Porridges are also easily digestible and can be prepared in a variety of ways, making them easy to incorporate into the infant diet; this is of particular importance as their high mineral and vitamin content make them invaluable dietary components for young children [11,12]. Recent studies indicate high rates of micronutrient deficiencies among children worldwide, with over 50% being estimated as being deficient in at least one essential nutrient. Micronutrients such as zinc, iron, copper, and manganese play a crucial role in child development, especially in the first years of life, when growth is rapid. Their deficiency can lead to significant developmental delays, weakened immune response, and poor health in children [13,14]. However, excessive consumption can lead to adverse effects, including hepatotoxicity (Fe, Cu), neurological development disorders (Mn), and mineral imbalance (Zn) [15,16].

Despite the existence of existing guidelines and quality controls for products intended for children and infants, a growing call has emerged for the implementation of more stringent requirements for such products. This phenomenon can be attributed to a number of factors:

A. There have been reports of elevated concentrations of noxious substances in products designed for infants and young children [17,18,19].
The adverse effects of inorganic contamination exposure in infants and children may include anemia, nephrotoxicity, developmental and reproductive toxicity, lower intelligence quotient (IQ), and neurotoxic effects. The underlying causes of this situation have been identified as inadequate testing practices, lenient standards, and limited oversight of some of the largest infant food manufacturers. Among the most hazardous of these elements are lead, cadmium, and mercury [16,20,21,22,23,24,25,26].
Lead has been shown to disrupt iron metabolism by inhibiting the formation of heme, which can result in anemia. Furthermore, evidence suggests that it exhibits developmental neurotoxicity in children. Research has demonstrated that the accumulation of lead during childhood can have a detrimental impact on cognitive abilities in adulthood [27,28,29].
Cadmium has been observed to primarily accumulate in the kidneys, specifically within the proximal tubule cells, resulting in impaired functionality of these cells. Furthermore, exposure to this element during early childhood has been demonstrated to result in diminished IQ scores and an augmented likelihood of attention deficit hyperactivity disorder (ADHD) [16,29,30,31,32,33,34].
Mercury has been demonstrated to possess neurotoxic, nephrotoxic, and immunotoxic properties. The substance’s primary accumulation sites are the kidneys and liver. Prenatal exposure to mercury has been linked to abnormal neurological development and reduced IQ [35].
B. The underdeveloped bodies of infants and young children render them particularly vulnerable to the harmful effects of minimal quantities of noxious substances.
The physiological processes of infants and young children differ from those of adults. This phenomenon can be attributed to several factors, including incomplete organ development. For instance, the stomach capacity of a six-month-old child is approximately 11 times smaller than that of an adult. Full liver function is not achieved until the age of two, and kidney filtration in young children is only 30–40% developed. Consequently, there is reduced enzyme secretion. The majority of digestive enzymes reach optimal activity by 6–7 months of age, while pancreatic amylase and pepsin only reach optimal activity at around 11–12 months of age. This results in reduced hydrochloric acid production in the stomach. This contributes to particular sensitivity to hazardous substances, including toxic elements [16,18,34,35,36,37,38].
C. The capacity of elements to form compounds within the body is significant.

The properties of trace elements, particularly those that are toxic, are as follows:

- Show a particular affinity for binding to thiol groups of proteins, inhibiting the production of over 200 enzymes in the human body.
- Can affect cells through mimicry. They can attach themselves to physiological sites that are normally reserved for essential elements, thereby disrupting normal biochemical and/or physiological functions
- Can act as catalysts for redox reactions with oxygen or other endogenous oxidants, causing oxidative modifications of proteins and DNA [16,35].

The formation of these compounds has the effect of deactivating important enzymes, thereby limiting the absorption of essential nutrients and, consequently, intensifying their harmful effects [16,17,18,19,20,21,22,23].

In view of the increasing consumption of dairy-free infant porridges and the limited availability of monitoring data for these products on the Polish market, further investigation of their mineral composition and potential health implications appears justified. Previous research indicates that cereal-based complementary foods may simultaneously contribute to the intake of essential trace elements and represent a potential source of exposure to toxic metals in infants. Nevertheless, data regarding dairy-free infant porridges with diverse cereal compositions remain limited.

Accordingly, the aim of this study was to assess the content of selected essential (Zn, Fe, Mn, and Cu) and potentially harmful (Pb, Cd, and Hg) trace elements in commercially available dairy-free infant porridges intended for children aged six months and older. Furthermore, the study sought to estimate the contribution of a single recommended serving of these products to the recommended daily allowance (RDA) or adequate intake (AI) of essential elements, as well as to evaluate potential health risks associated with exposure to toxic elements using estimated daily intake (EDI).

Based on existing evidence and the applied experimental approach, it was assumed that:

(1) the content of essential trace elements in dairy-free infant porridges may vary depending on their cereal-based raw material composition;
(2) a single serving of dairy-free infant porridge is expected to contribute to the dietary requirements for selected essential trace elements in infants, while remaining below established tolerable upper intake levels; and
(3) the concentrations and estimated daily intake of lead, cadmium, and mercury in these products are likely to remain within current European safety limits and are unlikely to pose a significant health risk to infants.

## 2. Materials and Methods

### 2.1. Research Material

The research material consisted of commercially available instant porridges intended for infants over six months of age. The selected products were dairy-free and did not contain any added flavors, aromas, or sweeteners (Table 1). The authors focused on popular dairy-free porridges available in Polish stationary and online stores. A total of seven distinct types of porridge (P1–P7, Table 1) were identified. Each type was sampled in five replicates over three time periods (different batch numbers).

#### 2.1.1. Criteria for the Selection of Infant Porridges

The selection of infant porridges for the present study was conducted using clearly defined inclusion and exclusion criteria to ensure the homogeneity of the research material and the relevance of the results to infant nutrition and food safety assessment.

The analyzed products met all of the following criteria:

- commercially available cereal-based porridges intended for infants aged six months and older, as declared by the manufacturer;
- dairy-free formulations, excluding milk proteins and milk-derived ingredients;
- products without added flavorings, sweeteners, fruits, herbs, or other functional additives, to minimize potential confounding sources of trace elements;
- porridges based exclusively on cereal grains or cereal-derived raw materials, including single-grain and multi-cereal compositions;
- products available on the Polish retail market, both in stationary and online stores, at the time of sampling;
- porridges requiring preparation by reconstitution with water, ensuring comparability of serving size and intake estimates.

Products were excluded from the study if they met any of the following conditions:

- porridges with unclear or unspecified age recommendations;
- products containing added milk, infant formula, or protein-rich ingredients;
- porridges enriched with fruit, vegetables, herbs, cocoa, or other non-cereal ingredients that could significantly affect trace element content;
- special-purpose products, including medical foods, therapeutic porridges, or products intended for infants with specific medical conditions;
- products marketed primarily as snacks or instant desserts rather than staple complementary foods.

#### 2.1.2. Sampling Strategy

A total of seven different types of dairy-free infant porridges were selected. Each product was sampled in five replicates, collected from three different production batches at different time points to account for potential batch-to-batch variability (Table 1).

### 2.2. Research Methods

#### 2.2.1. Chemical Analyses

The selected porridges were divided into 0.4 ± 0.001 g samples using a WPS 360/C balance (RADWAG, Radom, Poland) and mineralized in an MDS-2000 microwave oven (CEM Corp., Matthews, NC, USA) using 5 mL of 65% nitric acid and 1 mL of 30% hydrogen peroxide. To guarantee the quality of the analyses performed, all reagents were of the highest purity (Merck KGaA, Darmstadt, Germany). The deionized water used was prepared using a Barnstead EASYpure UV deionizer (0.05 μS/cm Barnstead™ GenPure™ Pro, Thermo Scientific, Hennigsdorf, Germany). These samples were accompanied by those prepared with reference materials (CRM NIST 1643f, Gliwice, Poland and CRM NIST 2383a, Gaithersburg, MD, USA) and blank (reagent) samples.

Mercury content was determined using a DMA-1 mercury analyzer (MILESTONE Srl, Bergamo, Italy) by atomic absorption spectrometry (AAS). The remaining elements were determined by inductively coupled plasma emission spectrometry (ICP-AES) in a Jobin Yvon JY-24 (ISA Jobin Yvon, Paris, France) apparatus equipped with a Meinhard TR 50-C1 nebulizer (Meinhard Glass Products, Ventura, CA, USA). Device settings included a generator frequency of 40.68 MHz, an output power of 1000 W, and the use of argon as the plasma gas (12.0 L/min), auxiliary gas (1.0 L/min), and nebulizer gas (1.1 L/min).

The quality of the selected analytical methods was verified based on the determination of the limit of detection (LOD) and limit of quantification (LOQ), recovery, and precision. The LOD and LOQ values were determined by blind deviations multiplied by three (LOD) or by designation (LOQ). The respective LOD and LOQ limits (μg/kg) were as follows: Zn—2.6, 8.7; Fe—3.1, 10.3; Mn—1.1, 3.7; Cu—3.2, 10.7; Pb—1.01, 3.37; Cd—0.09, 0.31; Hg—0.07, 0.23.

The quality of the analysis was verified based on the calibration coefficient values for every 12 samples. The acceptable calibration coefficient value was assumed to be ≥0.995. The accuracy of the analytical procedure was confirmed using certified reference material (INCT-SBF-4). The respective recovery values and coefficients of variation (CV) for the analyzed elements were as follows: Zn—98.3%, 2.7%; Fe—98.5%, 3.9%; Mn—101.2%, 3.1%; Cu—95.4%, 4.6%; Pb—96.6%, 3.5%; Cd—98.0%, 3.7%; Hg—99.1%, 2.0%.

#### 2.2.2. Calculating the Coverage of the Recommended Daily Allowance (RDA) and Optimal, i.e., Adequate, Intake (AI) of Elements by a Portion of Porridge

The degree of coverage of the recommended daily allowance (RDA) of Zn, Fe, and Cu, and the adequate intake (AI) of manganese when consuming one serving of porridge was determined according to applicable Polish standards [39].

#### 2.2.3. Calculation of the Health Risk Associated with Exposure to the Elements Supplied by a Portion of Porridge

Health risk was determined based on the following factors:

- EDI—estimated daily intake

EDI = (MS × C)/BM [µg/kg body weight/day]

MS—amount of porridge in a single serving, as recommended by the manufacturer (Table 1); C—element content in the analyzed porridges (mg/kg); BM—mean body weight of a child at seven months of age (8.25 kg), with this value determined according to percentile charts for children.

- ADI—Acceptable Daily Intake [40]

ADI (%) = (EDI × 100)/(ADI × BW)

ADI—for: Cu—0.57 mg/kg bw; Pb—0.0036 mg/kg bw; Cd—0.001 mg/kg bw; Hg—0.0001 mg/kg bw; BW—mean body weight of a child at seven months of age (8.25 kg, value determined according to percentile charts for children).

- UL—Tolerable Upper Intake Level [41]

UL (%) = (MS × C × 100)/UL

MS—amount of porridge in one serving, as recommended by the manufacturer (Table 1); C—element content in the analyzed porridges (mg/kg); UL—for Zn—5 mg/day; for Fe—40 mg/day.

#### 2.2.4. Statistical Analysis

Statistical analyses were performed using the Statistica 13.3 PL package (StatSoft, Kraków, Poland). Significant differences in trace element content between different types of porridge were identified using one-way analysis of variance (ANOVA) at a significance level of *p* < 0.05. The significance of differences between groups was tested using Duncan’s post hoc test (*p* < 0.05).

## 3. Results

### 3.1. Essential Trace Elements

**Figure 1:**
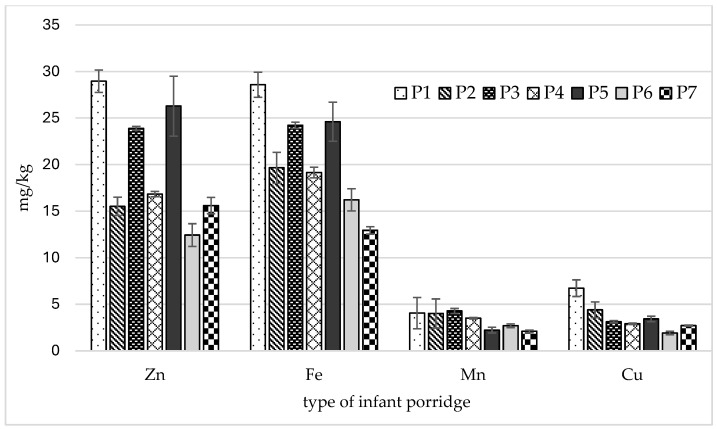
Mean values and standard deviations of analyzed elements.

The analyzed porridges contained varying amounts of essential elements (Figure 1). Zinc content varied considerably between porridge types, ranging from 11.6 mg/kg (P6) to 30.3 mg/kg (P1). The differences found were statistically significant (Table 2).

The iron content in the tested infant porridges ranged from 12.6 mg/kg (P7) to 29.2 mg/kg (P1), with almost all differences between types being statistically significant (Table 2). The mean manganese content for all porridges was 3.27 mg/kg (ranged from 1.87 to 4.57 mg/kg), with significantly higher levels being found in the P3 groats (4.57 mg/kg) than the other samples (Table 2). The copper content in the analyzed samples ranged from 1.74 mg/kg to 7.04 mg/kg. The highest copper content was detected in sample P1. Significantly higher copper levels were noted in samples P1 and P2 compared to the others (Table 2).

#### 3.1.1. The Degree to Which Infant Porridge Covers the Daily Requirement (RDA) and the Adequate Intake (AI) of Essential Nutrients

A single serving of infant porridge constituted 8.3% to 21.3% of the daily requirement for zinc, with the highest value recorded in sample P1 and the lowest in sample P6 (Table 3). However, a serving accounted for significantly less of the RDA for iron, ranging from 2.6% (P7) to 5.7% (P1) (Table 3).

None of the porridges provided more than 6% of the recommended daily intake of iron, indicating a marginal contribution in this regard.

A recommended portion of porridge provided 12.76% (P6) to 49.36% (P1) of the recommended intake of copper, but less than 15% of manganese (Table 3).

#### 3.1.2. Estimated Health Risks for Infants Associated with the Consumption of Essential Elements from a Serving of Porridge

The estimated daily intake (EDI) of zinc from a single serving of infant porridge ranged from 27.4 µg/kg bw/day (P6) to 63.7 µg/kg bw/day (P1) (Table 4); zinc content was found to vary depending on the type of product, which may result from the raw material composition of the individual mixtures. The EDI of iron ranged from 28.5 µg/kg bw/day (P7) to 62.9 µg/kg bw/day (P1) for a portion of porridge (Table 4), while the EDI of manganese did not exceed 9.5 µg/kg bw/day (P3), and the EDI of copper ranged from 4.21 µg (P6) to 14.81 µg/kg bw/day (P1) (Table 4).

### 3.2. Harmful Trace Elements

**Figure 2:**
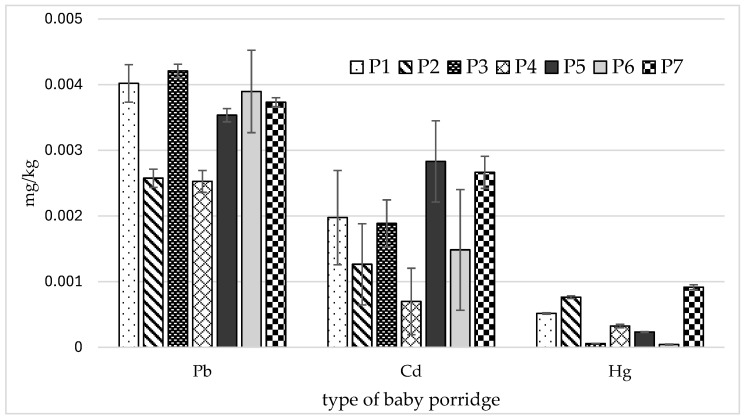
Mean values and standard deviations of the analyzed harmful elements.

Small amounts of harmful elements were identified in the tested porridges (Figure 2).

The lead content in the tested samples of baby porridges was very low and did not exceed 0.005 mg/kg, with significantly lower amounts being observed in samples P2 and P4 (Figure 2). Similar levels of cadmium (Cd) were noted, ranging from <0.001 mg/kg to 0.003 mg/kg depending on the sample; however, in most cases, these differences were not statistically significant. Small amounts of mercury were also identified (<0.001–0.001), with significant differences found between samples (Table 5).

#### Estimated Health Risks for Infants Related to the Intake of Harmful Elements

The estimated daily intake of lead contained in one serving of infant porridge did not exceed 0.01 µg/kg bw/day (Table 4), with only small differences in EDI observed between samples. Due to its toxicity and potential to accumulate, the presence of this element, even in trace amounts, requires special attention; this is particularly important for infants. The EDI of cadmium associated with a single serving of porridge was also very low. The maximum EDI of mercury was 0.002 µg/kg bw/day (P2 and P7) (Table 4), i.e., very low, which again is particularly important when ensuring food safety for children.

## 4. Discussion

### 4.1. Essential Trace Elements

The analyzed infant porridges demonstrated significant differences in trace element content, which can be attributed to their varied compositions, which were dominated by combinations of organic and non-organic cereal grains, groats and flours, and possible mineral additives [42,43]. Indeed, Sinkovič et al. [44] report significant differences in Zn, Fe, and Cu content between flour and whole spelt depending on the plant genotype and fraction.

The highest zinc values were recorded in sample P1, which contained only organic millet and oat groats. Both of these ingredients naturally contain relatively high amounts of zinc, especially in their unrefined form. Similar levels of Zn were found in sample K5 (100% buckwheat) and P3 (mix of five wholegrain cereals), indicating that higher zinc levels can be present in porridge derived from different types of grains, especially in unhulled or minimally processed forms. A similar relationship was observed by Pecev-Marinković et al. [45], who indicated that wholegrain cereal products are richer in Zn and other micronutrients than their purified counterparts. In turn, significantly lower levels were noted in rice and corn porridges (P6, P7); this is in line with previous findings that these cereals are poor in zinc [46,47].

The recommended daily intake (RDA) of zinc is 3 mg for children aged both six to twelve months and one to three years [39,48,49]. Unfortunately, growing numbers of infants and young children exhibit zinc deficiency, with levels being low enough to inhibit growth and in some populations [50]. In such situations, zinc supplementation is recommended, as zinc deficiency between six months and five years of age has been found to increase the risk of death from diarrhea and pneumonia [51,52,53,54]. It is therefore generally advisable to provide dietary enrichment to ensure infants meet their zinc requirement [55]. However, excessive zinc intake can result in gastrointestinal disturbances and impaired absorption of other essential elements, such as copper and iron [56,57].

In the case of iron, the highest values were noted in P1, P3 and P5, i.e., porridges with a complex composition based on whole-grain cereal products. Buckwheat, spelt, rye and barley are relatively rich in iron, which may explain their observed advantage over porridges based on rice or corn (P6, P7), which showed the lowest iron content. Similar differences were also noticed by Kiani et al. [58], who report that while millet and cereal porridges provide higher levels of iron than rice products, they still fail to fully meet the daily requirement. This is confirmed by our present data indicating that all tested samples make a relatively poor contribution to recommended iron intake; hence, porridges should not be considered as the main source of iron in the infant diet [59].

After six months of age, following the depletion of the iron stores accumulated in utero and the accelerated pace of physical and neurological development, an infant’s iron requirements increase significantly. Indeed, the recommended daily intake continues to grow from 7 mg for infants aged six to twelve months to 11 mg for children aged one to three years [39,60,61]. Iron is involved in the formation of hemoglobin, enabling the essential transport of oxygen to tissues during periods of intensive growth [45]. It also participates in the myelination of neurons and the synthesis of neurotransmitters; as such, its deficiency can lead to impaired cognitive and neurological development [58].

Iron insufficiency is one of the most common nutritional deficiencies worldwide. In infants and young children, deficiency can lead to anemia, impaired cognitive and motor function, and increased susceptibility to infection [62,63]. However, excessive iron intake can lead to other issues such as gastrointestinal disorders, constipation and, potentially, liver and kidney damage [64,65]. Therefore, it is important that iron consumption should be in line with recommendations, and should not exceed the recommended maximum intake [50]. For infants, it is recommended they regularly consume meat, as a source of heme iron, and beans and fortified cereal products, for non-heme iron [66]. For those receiving a plant-based diet, it should also be noted that the absorption of non-heme iron is significantly increased by vitamin C consumption [58].

The highest manganese content was found in samples P1, P2, and P3, i.e., porridges containing oats, spelt, and barley, confirming previous reports that these cereals are particularly rich in manganese [44]. Significantly lower levels were noted in samples P5, P6, and P7, based mainly on buckwheat, corn, and rice, and in samples K5, K6, and K7, based mainly on buckwheat, corn, and rice. Samples P5, P6, and P7, based mainly on buckwheat, corn, and rice, contained significantly less of this element. Manganese is involved in the production of enzymes necessary for the metabolism of amino acids, lipids, and carbohydrates, as well as in the regulation of blood sugar levels [67,68]. In the infant diet, the most common source is consumption of whole grains.

Manganese deficiency in infants and young children can lead to neurodevelopmental disorders, including cognitive and motor deficits, as well as skeletal abnormalities such as bone deformities and reduced bone density [69,70]. However, excessive consumption can also have negative effects, ranging from neurological symptoms such as tremors and cognitive impairment, to liver and kidney damage [71]. According to dietary recommendations for the Polish population, the recommended daily intake (AI) of manganese is 0.6 mg for infants aged six to twelve months and 1.2 mg for children aged one to three years [39].

Another important component of the human diet is copper, an element readily available from oats, especially their whole-grain form. However, exceeding the norms for Cu may be associated with long-term metabolic burden, especially in infants and young children [39,40,41]. Copper participates in various metabolic processes, including neurotransmitter synthesis and antioxidant defense mechanisms; it also acts as a cofactor for enzymes involved in energy metabolism, whose inhibition due to copper insufficiency leads to impaired mitochondrial function and increased oxidative stress [72]. Copper is also a component of ceruloplasmin, which plays a significant role in iron transport and utilization [73].

According to current Polish guidelines, the recommended daily copper intake for children aged around six months is 0.3 mg [39]. It is easily obtained from nuts, whole grains, and some types of meat [74], as well as various seeds, lentils, and seafood [75]. Higher levels of dietary copper have been shown to increase the activity of lipases and other digestive enzymes, which translates into more efficient digestion [76]. Copper deficiency can disrupt the growth hormone axis, leading to impaired somatic development [77], and may contribute to the development of heart issues such as cardiomyopathy [72,78]. Infants receiving long-term parenteral nutrition are particularly vulnerable to deficiencies, as standard nutritional solutions often lack sufficient amounts of Cu; this can lead to serious complications, such as hematological and neurological disorders and delayed bone development. Therefore, it is important to regularly monitor the level of this copper in the body and provide appropriate supplementation in this vulnerable group of patients [78,79,80,81].

Our findings indicate positive correlations between the levels of iron, zinc, and copper, confirming that they derive from common dietary sources, mainly wholegrain products. This mutual correlation can also be attributed to their similar mechanisms of absorption and transport in the body [44].

Duncan’s test, in turn, confirmed significant differences between samples for almost all elements. The greatest variation was observed for copper and zinc, which may indicate inconsistent approaches to enrichment and raw material selection between producers. Similar observations were made in a study of the elemental composition of cereals and flours by Kraska et al. [82]; while the overall quality of the raw materials was found to be good, with low levels of toxic elements, large differences in content were found, which were attributed to the origin of the raw material and the milling process. These differences are particularly important from the point of view of consumer safety, as regular consumption may lead to the accumulation of heavy metals or excess of micronutrients, even if the foodstuffs themselves do not exceed standards. While little research has been performed on the content of harmful ingredients in baby products, isolated reports indicate a risk of contamination, stemming from the addition of inter alia rice or fish [40,46].

### 4.2. Harmful Trace Elements

Regarding the harmful components, lead accumulates in the body and its presence is associated with impaired cognitive development, lower intelligence quotient (IQ), and behavioral problems; children with high levels of lead are also at greater risk of stunted growth and attention deficit hyperactivity disorder (ADHD). Lead exposure can also affect the cardiovascular, renal, and reproductive systems and is associated with anemia and other hematological disorders [83,84,85,86]. Children are particularly vulnerable to lead toxicity because of their developing nervous systems, which make them more susceptible to its effects. Food and beverages can become contaminated with lead through contact with contaminated soil or water, which is believed to represent the main source of lead in cereals and other plant products. Contamination can also originate from anthropogenic sources, such as packaging or manufacturing processes [83,84,87].

Infants and young children are also at increased risk of cadmium exposure due to their rapid growth and development, as well as higher food consumption relative to body weight. Food may become contaminated with cadmium through contaminated soil, water and air, as well as poor agricultural practices [88,89,90]. Excess cadmium in the body can increase the risk of osteoporosis and bone loss, even at low levels of exposure. It can also negatively impact the cardiovascular system, potentially contributing to the development of hypertension and other cardiovascular diseases. Excess cadmium also leads to an increased risk of kidney disease and has been linked to the development of certain types of cancer, such as lung and prostate cancer [88,91].

Mercury can disrupt the proper functioning of the nervous system by disrupting the structure and function of neurons, impairing neurotransmitter systems, and inducing oxidative stress. Exposure to mercury, particularly during critical periods of brain development, can lead to neurological and cognitive disorders, including delays in motor and language development, as well as attention and memory deficits [92,93]. Prenatal and early childhood exposure to mercury, particularly in the form of methylmercury, has been associated with adverse neurological outcomes such as reduced IQ, impaired language and memory skills, and increased risk of autism spectrum. Mercury exposure may also affect the cardiovascular system, leading to an increased risk of cardiovascular diseases, including hypertension. Long-term exposure can cause neurological symptoms such as tremors, ataxia, and sensory disturbances, as well as kidney and liver damage [92,93,94,95].

### 4.3. Study Limitations and Implications for Future Research

Despite providing comprehensive data on the trace element composition of dairy-free infant porridges, several limitations related to dietary exposure assessment should be acknowledged. The estimation of dietary intake in the present study was based on manufacturer-declared portion sizes, pediatric dietary recommendations, a representative infant body weight, and commonly applied risk assessment indicators, including estimated daily intake (EDI), percentage of recommended daily allowance or adequate intake (RDA/AI), and intake normalized to body weight. While this approach enables standardized comparison across products, it may not fully reflect real-life feeding practices.

Actual portion sizes consumed by infants may vary depending on age, developmental stage of complementary feeding, appetite, and adherence to pediatric guidelines. Moreover, the dietary exposure assessment focused on the intake derived from a single product and did not account for cumulative exposure resulting from the consumption of other complementary foods or infant formulas during the same day. As a result, total daily intake of essential and potentially harmful trace elements may differ from the estimates presented in this study.

In addition, dietary exposure calculations were performed for a single representative age corresponding to infants at approximately seven months of age. Although this approach is consistent with established risk assessment methodologies, it does not capture age-related variability in body weight, portion size, and nutritional requirements among infants aged six months and older. The availability of comparable experimental data stratified by infant age groups remains limited, which precluded a direct comparison across age categories within the scope of the present study.

Future research should therefore aim to refine dietary exposure assessment by integrating age-specific pediatric feeding guidelines with portion sizes declared on product labels and realistic consumption scenarios. In particular, categorizing infants into two representative age groups, such as early complementary feeding (6–8 months) and later infancy (9–12 months), could improve the precision of estimated daily intake (EDI), percentage of RDA/AI coverage, and intake expressed per kilogram of body weight. Additionally, incorporating cumulative dietary exposure from multiple complementary foods would allow for a more comprehensive evaluation of nutritional adequacy and potential health risks associated with trace element intake in infants.

## 5. Conclusions

The present study provides data on the content of selected essential and potentially harmful trace elements in dairy-free infant porridges available on the Polish market and their contribution to dietary exposure in infants aged six months and older. When assessed using manufacturer-declared portion sizes, pediatric feeding recommendations, and body weight-normalized intake indicators, the analyzed products were found to contribute measurably to the intake of essential trace elements, particularly copper and zinc. At the same time, the concentrations and estimated daily intake (EDI) of lead, cadmium, and mercury associated with the consumption of a single recommended serving remained low and did not exceed current regulatory reference values. These findings suggest that, under typical consumption scenarios, dairy-free infant porridges may represent a nutritionally relevant component of complementary feeding while posing a limited risk with respect to exposure to the analyzed toxic elements. Nevertheless, given variability in infant age, body weight, portion size, and overall dietary patterns, the results should be interpreted with caution. Further studies incorporating age-specific intake scenarios, cumulative dietary exposure, and broader product ranges would help to refine the assessment of nutritional adequacy and safety of dairy-free infant porridges.

## Figures and Tables

**Table 1:** Characteristics of infant’s porridges purchased for testing.

Type of Porridge	Product Composition		Recommended Amount in One Serving
P1	ecological millet groats ecological oat groats	55% 45%	22 g
P2	whole grain oat flour rice flour antioxidant (ascorbic acid), thiamine	51% 49%	22 g
P3	organic peeled buckwheat groats organic peeled barley organic whole grain spelt flour organic whole grain rye flour organic whole grain oat flour	20.8% 20.8% 20.8% 20.8% 16.8%	20 g
P4	organic whole grain spelt flour organic heat-treated buckwheat groats organic round grain rice groats	41.7% 41.7% 16.6%	20 g
P5	organic, heat-treated buckwheat groats	100%	20 g
P6	organic corn flour ecological whole grain oat flour	58.3%, 41.7%	20 g
P7	whole grain rice flour corn flour whole grain millet flour thiamine	70% 20% 10%	22 g

**Table 2:** Significant differences in the content of selected essential elements between the analyzed porridge types (Duncan’s test).

**Zn**	**P1**	**P2**	**P3**	**P4**	**P5**	**P6**	**Fe**	**P1**	**P2**	**P3**	**P4**	**P5**	**P6**
**P2**	**0.00003**						P2	**0.00007**					
**P3**	**0.00314**	**0.00010**					P3	**0.00209**	**0.00116**				
**P4**	**0.00007**	0.37639	**0.00030**				P4	**0.00004**	0.65097	**0.00063**			
**P5**	0.07009	**0.00004**	0.10202	**0.00010**			P5	**0.00312**	**0.00078**	0.74094	**0.00042**		
**P6**	**0.00003**	**0.04037**	**0.00004**	**0.00937**	**0.00003**		P6	**0.00003**	**0.00996**	**0.00007**	**0.01930**	**0.00004**	
**P7**	**0.00004**	0.95222	**0.00012**	0.38251	**0.00007**	**0.04377**	P7	**0.00003**	**0.00011**	**0.00004**	**0.00017**	**0.00003**	**0.01047**
**Mn**	**P1**	**P2**	**P3**	**P4**	**P5**	**P6**	**Cu**	**P1**	**P2**	**P3**	**P4**	**P5**	**P6**
**P2**	0.84170						P2	**0.00018**					
**P3**	0.25964	0.21133					P3	**0.00007**	**0.00016**				
**P4**	**0.01764**	**0.02086**	**0.00223**				P4	**0.00004**	**0.00008**	0.38017			
**P5**	**0.00004**	**0.00007**	**0.00003**	**0.00010**			P5	**0.00009**	**0.00085**	0.20475	0.05099		
**P6**	**0.00007**	**0.00010**	**0.00004**	**0.00105**	**0.02714**		P6	**0.00003**	**0.00003**	**0.00025**	**0.00090**	**0.00006**	
**P7**	**0.00003**	**0.00004**	**0.00003**	**0.00007**	0.52651	**0.00977**	P7	**0.00003**	**0.00004**	0.11424	0.40262	**0.01182**	**0.00350**

**bold**—significant differences (*p* < 0.05).

**Table 3:** Coverage of the recommended daily allowance (RDA) and adequate intake (AI) of nutrients by a single portion of porridge.

Type of Porridge	Zn	Fe	Cu	Mn
	RDA = 3 mg/24 h *	RDA = 11 mg/24 h *	RDA = 0.3 mg/24 h *	AI = 0.6 mg/24 h *
	% RDA	% RDA	% RDA	% AI
P1	21.2	5.7	49.4	14.9
P2	11.4	3.9	32.3	14.7
P3	15.9	4.4	20.8	14.3
P4	11.2	3.5	19.4	11.7
P5	17.5	4.5	22.8	7.4
P6	8.3	2.9	12.8	9.0
P7	11.4	2.6	19.9	7.7

* Polish recommendations [39].

**Table 4:** Estimated Daily Intake (EDI) of essential elements contained in one portion of infant porridge.

Type of Porridge	EDI [µg/kg bw/Day]						
	Zn	Fe	Mn	Cu	Pb	Cd	Hg
P1	63.7	62.9	8.92	14.81	0.009	0.004	0.001
P2	34.1	43.3	8.83	9.69	0.006	0.003	0.002
P3	52.6	53.3	9.42	6.86	0.009	0.004	<0.001
P4	37.0	42.1	7.73	6.41	0.006	0.002	0.001
P5	57.8	54.1	4.90	7.53	0.008	0.006	0.001
P6	27.4	35.7	5.95	4.21	0.009	0.003	<0.001
P7	34.3	28.5	4.63	5.98	0.008	0.006	0.002

**Table 5:** Significant differences in the content of selected harmful elements between samples (Duncan’s test).

**Pb**	**P1**	**P2**	**P3**	**P4**	**P5**	**P6**	**Cd**	**P1**	**P2**	**P3**	**P4**	**P5**	**P6**
**P2**	0.0001 *						P2	0.2049					
**P3**	0.4216	**0.0000**					P3	0.8585	0.2523				
**P4**	**0.0001**	0.8366	**0.0000**				P4	**0.0335**	0.2721	**0.0427**			
**P5**	0.0694	**0.0010**	**0.0173**	**0.0009**			P5	0.1218	**0.0125**	0.0983	**0.0017**		
**P6**	0.5981	**0.0001**	0.2148	**0.0001**	0.1548		P6	0.3614	0.6635	0.4301	0.1532	**0.0260**	
**P7**	0.2546	**0.0003**	0.0745	**0.0002**	0.3991	0.4887	P7	0.1849	**0.0214**	0.1561	**0.0028**	0.7418	**0.0436**
**Hg**	**P1**	**P2**	**P3**	**P4**	**P5**	**P6**							
**P2**	**0.0002**												
**P3**	**0.0001**	**0.0000**											
**P4**	**0.0002**	**0.0001**	**0.0001**										
**P5**	**0.0001**	**0.0001**	**0.0002**	**0.0002**									
**P6**	**0.0000**	**0.0000**	0.3382	**0.0001**	**0.0001**								
**P7**	**0.0001**	**0.0002**	**0.0000**	**0.0001**	**0.0000**	**0.0000**							

* bold—significant differences (*p* < 0.05).

## Data Availability

The raw data supporting the conclusions of this article will be made available by the authors on request.

## Abbreviations

The following abbreviations are used in this manuscript:

ICP-AES	Inductively Coupled Plasma Emission Spectrometry
Hg-AAS	Mercury-Atomic Absorption Spectrometry
RDA	Recommended Daily Allowance
AI	Adequate Intake
